# Laryngeal Reinnervation Using Ansa Cervicalis for Thyroid Surgery-Related Unilateral Vocal Fold Paralysis: A Long-Term Outcome Analysis of 237 Cases

**DOI:** 10.1371/journal.pone.0019128

**Published:** 2011-04-29

**Authors:** Wei Wang, Donghui Chen, Shicai Chen, Ding Li, Meng Li, Siwen Xia, Hongliang Zheng

**Affiliations:** Department of Otorhinolaryngology-Head and Neck Surgery, Changhai Hospital, Second Military Medical University, Shanghai, China; University of Southern California, United States of America

## Abstract

**Objective:**

To evaluate the long-term efficacy of delayed laryngeal reinnervation using the main branch of the ansa cervicalis in treatment of unilateral vocal fold paralysis (UVFP) caused by thyroid surgery.

**Summary of Background Data:**

UVFP remains a serious complication of thyroid surgery. Up to now, a completely satisfactory surgical treatment of UVFP has been elusive.

**Methods:**

From Jan. 1996 to Jan. 2008, a total of 237 UVFP patients who underwent ansa cervicalis main branch-to-recurrent laryngeal nerve (RLN) anastomosis were enrolled as UVFP group; another 237 age- and gender-matched normal subjects served as control group. Videostroboscopy, vocal function assessment (acoustic analysis, perceptual evaluation and maximum phonation time), and electromyography were performed preoperatively and postoperatively. The mean follow-up period was 5.2±2.7 years, ranging from 2 to 12 years.

**Results:**

Analysis of videostroboscopic findings indicated that the glottic closure, vocal fold edge, vocal fold position, phase symmetry and regularity were significantly improved in the UVFP group (*P*<0.001, postoperative *vs.* preoperative). The postoperative parameters of vocal function were also significantly improved in the UVFP group (*P*<0.001) and showed no statistical differences compared to the control group (*P*>0.05, respectively). Postoperative laryngeal electromyography confirmed successful reinnervation of laryngeal muscle.

**Conclusions:**

Delayed laryngeal reinnervation with the main branch of ansa cervicalis is a feasible and effective approach for treatment of thyroid surgery-related UVFP; it can restore the physiological laryngeal phonatory function to the normal or a nearly normal voice quality.

## Introduction

Vocal fold paralysis (VFP) is one of the most severe complications caused by thyroid surgery-associated recurrent laryngeal nerve (RLN) injury [1]. The incidence of the permanent VFP was 1%–2% despite of the improvements made in surgical techniques and application of intra-operative nerve monitoring [2], [3], [4]. Unilateral vocal fold paralysis (UVFP) is characterized by varying degrees of hoarseness, poor coughing effort, and occasional aspiration, which impair the quality of life of the patients. Dissatisfied patients often seek legal redress that can lead to costly, time-consuming litigation with the surgeons. Therefore, to properly handle thyroid surgery-related UVFP is also an important issue for these surgeons.

If RLN is unexpectedly injured during operation or the involved RLN is removed due to resection of thyroid malignancy, direct RLN anastomosis, as well as free nerve grafting or ansa cervicalis-to-RLN anastomosis, could be the choices for treatment of UVFP [5], [6], [7]. However, intra-operative RLN injury is often inapparent to the surgeon during thyroid surgery, and can not be diagnosed until VFP-related symptoms develop postoperatively. For RLN injury, some surgeons have suggested that early surgical intervention can achieve better voice outcomes. However, most UVFP patients can rehabilitate vocal function by spontaneous nerve regeneration, so at least six months are generally allowed for possible spontaneous recovery or compensation from the contralateral vocal fold in clinic. Only in cases with dissatisfactory recovery should surgical intervention be considered. Presently surgical procedures including vocal fold injection, thyroplasty, arytenoid adduction, and laryngeal reinnervation can be expected to restore vocal function of symptomatic UVFP. Although improved voice quality can be obtained by vocal fold injection, thyroplasty and arytenoid adduction, these static procedures cannot prevent the atrophy of denervated laryngeal muscles. Theoretically, laryngeal reinnervation is an ideal approach that can restore the connection of motoneurons with denervated laryngeal muscles. Neurorrhaphy of the ansa cervicalis to RLN is one of the most popular laryngeal reinnervation procedures [8]. Anastomosis of a single branch of ansa cervicalis to RLN was re-introduced by Crumley, followed by Miyauchi, Oslon, Lee, Smith and other investigators [8], [9], [10], [11], [12]. Our previous anatomical study demonstrated that there is usually a main branch coming from the loop of ansa cervicalis, and it has two to three branches and innervates the inferior portions of the sternohyoid, sternothyroid and omohyoid muscles. The main branch almost has the same number of myelinated fibers and motor fibers to the number of the adductor branches of the RLN [13], [14]. The existence of the main branch of ansa cervicalis was later confirmed by Chhetri *et al* [15].

We firstly reported laryngeal reinnervation using main branch of ansa cervicalis in UVFP animal models in 1996, and satisfactory or good voice qualities were obtained by this procedure in a small sample of patients with UVFP [14], [16], [17]. The main branch of ansa cervicalis in our procedure possesses more motor fibers than a single branch. In our previous study, we observed significant improvement of voice quality which occurred abruptly 2–3 months after operation, and the voice quality kept improving until 6 months postoperatively. Lee and Lorenz et al. reported that delayed laryngeal reinnervation using ansa cervicalis was efficient in treating 46 UVFP patients, who were followed up to 18 months [8], [11]. In the present study, we included 237 thyroid surgery-related UVFP patients undergoing delayed laryngeal reinnervation using main branch of ansa cervicalis-to-RLN anastomosis, and evaluated the feasibility and efficacy of the procedure by assessing the long-term outcomes (at least 2 years). In addition, the critical surgical details of our procedure were also described to help the surgeons to handle the thyroid surgery-related UVFP.

## Materials and Methods

### Patient characteristics

This study was approved by the Institutional Review Broad of Second Military Medical University. The charts were reviewed on 256 patients who underwent the ansa cervicalis main branch-to-RLN anastomosis as a treatment for thyroid surgery-caused UVFP from Jan. 1996 to Jan. 2008. Nineteen patients were lost to follow-up. Consequently, 237 patients (59 males and 178 females, mean age 45.2 years, ranging 17–62 years) were enrolled as UVFP group. All clinical investigation was conducted according to the principles expressed in the Declaration of Helsinki. The informed consents were obtained from all patients involved in the present study. The consents were written and the ethics committees approved the consent procedure. A minimum of 6 months was allowed to elapse after known onset of the paralysis to permit possible spontaneous reinnervation or compensation. The median time of UVFP prior to surgery was 14.4 months (ranging 6–42 months). The follow-up period ranged 2–12 years, with a median of 5.2 years.

The control group included 237 age- and gender-matched normal subjects, who had no history of tobacco use, endotracheal intubation, neurological disease, laryngeal surgery or trauma, radiation exposure to head and neck, or laryngopharyngeal reflux. Furthermore, a complete videostroboscopic examination was performed to exclude any pathological findings that may lead to voice alterations in the control group.

### Operation procedure

**Figure 1:**
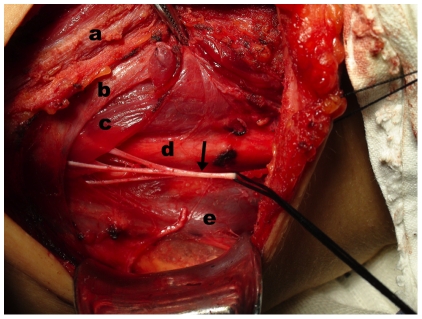
Intraoperative findings of the main branch of ansa cervicalis. The main branch of ansa cervicalis (arrow) which usually overlies the common carotid artery (d) or jugular vein (e), contains three sub-branches and innervates the inferior portions of the sternohyoid (a), omohyoid (b) and sternothyroid (c) muscles.

**Figure 2:**
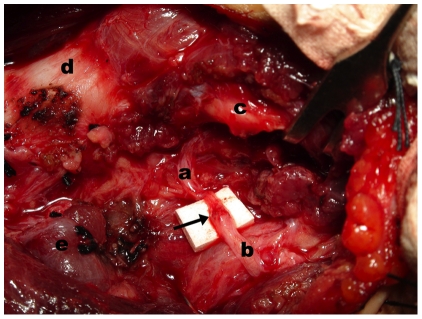
Intraoperative image of ansa cervicalis main branch-to-RLN anastomosis. Intraoperative image showing that the main branch of ansa cervicalis (b) was anastomosed to the distal stump of recurrent laryngeal nerve (a). c: inferior horn of thyroid cartilage; d: trachea; e: residual thyroid gland; arrow: anastomosis site of ansa cervicalis and recurrent laryngeal nerve.

Under general anesthesia, a horizontal oblique incision was made at the original incision of thyroid surgery or approximately 2 cm above the suprasternal notch. The fascia between the strap muscle and the sternomastoid muscle was opened on the paralyzed side. The internal jugular vein was exposed. The loop of the ansa cervicalis was usually seen through the fascia overlying the common carotid artery or jugular vein. This fascia was carefully incised and the nerve was traced distally. Usually, the main branch comes from the loop of ansa cervicalis. It has two to three sub-branches and innervates the inferior portions of the sternohyoid, sternothyroid and omohyoid muscles. (see Fig. 1). The main branch was then transected at the bifurcation and freely mobilized for preparation of anastomosis. The thyroid cartilage was engaged with a skin hook and gently rotated anteriorly, and the RLN was dissected retrogradely far enough to provide a tension-free anastomosis and then transected. Under an operating microscope, the nerve ends of distal RLN stump should be prepared with resection of scarred tissue back to fasicles. As described by Lee et al. [11], the distal RLN stump was trimmed to the minimal to promote early innervation. The external epineurium can be gently peeled to allow separation and identification of fascicles. The distal RLN stump was anastomosed to the main branch of ansa cervicalis using 3–5 epineurial stitches of nylon 11-0 thread (see Fig. 2). Care should be taken to avoid driving the needle or suture through the fascicles. Finally, the wound was closed in layers.

### Videostroboscopy

All patients were observed during “eee” phonation at a comfortable loudness and pitch as long as possible using a videostroboscope (wolf, model 5570) and the dynamic videos were recorded. Three experienced/senior laryngologists who had not performed any operation reviewed all of the videos. The videos were randomized and the reviewers were blinded to whether the videos were preoperative or postoperative. Our videostroboscopic rating method, a combination of the methods described by Lorenz and Rosen [11], [18], included the examination of glottal closure (0, complete; 1, slightly incomplete; 2, moderately incomplete; 3, severely incomplete); vocal fold position (0, midline; 1, paramedian; 2, intermediate; 3, lateral); vocal fold edge of paralyzed side (0, straight; 1, mildly bowing; 2, moderately bowing; 3, severely bowing); phase symmetry (0, normal; 1, mildly asymmetrical; 2, moderately asymmetrical; 3, severely asymmetrical); and regularity (0, normal; 1, mildly irregular; 2, moderately irregular; 3, severely irregular). The phase symmetry and regularity were considered as severely asymmetry and irregular if the voice was too poor for stroboscopic image. Consensus of the reviewers was reached on the visual appearance of the larynx.

### Vocal function assessment

Vocal function assessment included perceptual evaluation, acoustic analysis and maximum phonation time (MPT). Preoperative and postoperative voice samples which contain sustained vowels /a/ and connected speech samples, were subjected to perceptual evaluation and acoustic analysis. The recording equipment consisted of a digital audiotape recorder and a dynamic microphone (Tiger Electronics Inc., North Reading, MA, USA). Five laryngologists performed voice assessment using a perceptual rating scale (GRBAS) for voice quality and characteristics. The ratings were accomplished in a blinded fashion with patient voice samples arranged in a random manner. The listeners were asked to grade connected speech samples for overall grade, roughness, breathiness, asthenia and strain. This perceptual scale allows each listener to rate the voice quality on a scale (0 = normal, 1 = mild, 2 = moderate, 3 = severe) for each of the above parameters. The values were averaged among 5 listeners.

The acoustic parameters of sustained vowels /a/ were evaluated by Praat software (Version 5.1.12). The acoustic parameters involved mean noise to harmonics ratio (NHR) and measures of phonatory stability, namely, Jitter (local) and Shimmer (local). MPT (the duration of sustained phonation of the vowel /a/ after maximum inspiration) was measured preoperatively and postoperatively. MPT is generally thought to be an index of glottal efficiency.

### Electromyography (EMG)

EMG tracings were obtained from all patients before operation, while 209 patients consented to EMG postoperatively. A four-channel electromyograph and concentric needle electrodes (Dantec Counterpoint, Copenhagen, Denmark) were used for the EMG recording. To test for the proper needle position, the unaffected vocal fold was firstly examined. Electromyographic activities of bilateral thyroarytenoid (TA) muscles were recorded under the following two states: 1) when the patients breathed quietly with the relaxation of the body; 2) when the patients sustained the vowel /eee/ with the greatest exertion. One board-certified otolaryngologist performed EMG and a neurologist operated the EMG machine and interpreted the EMG results. The neurologist rated the voluntary motor-unit recruitment using the following scale: 0 = full interference, 1 = mixed interference, 2 = simple interference and 3 = without motor unit potential.

### Statistical Analysis

The data of acoustic analysis and MPT did not follow normal distribution and were presented as median (low quartile, upper quartile). Statistical difference between pre- and post-operative data of videostroboscopy, EMG, perceptual evaluation, acoustic analysis and MPT were analyzed using Wilcoxon's signed-rank test. And statistical difference in acoustic analysis and MPT were analyzed using Mann-Whitney U test between the UVFP group and control group. Intra-rater reliability in perceptual evaluation was analyzed by kappa coefficient. Statistical analysis adopted the software of SPSS 17.0 for Windows package ( SPSS Inc., Chicago, IL ). A *p* value less than 0.05 was considered as statistically significant.

## Results

### Videostroboscopic findings

**Figure 3:**
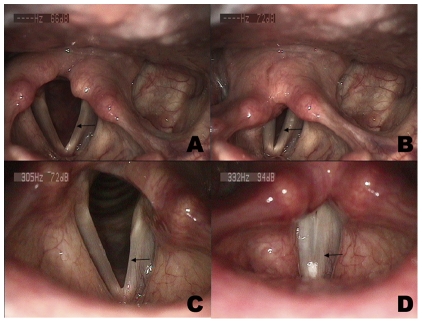
Videostoboscopic findings of a representative unilateral vocal fold paralysis (left). Preoperatively, the edge of the paralyzed vocal fold (arrow) appeared mildly bowed and vocal fold position was intermediate during inspiration (A), and a fully incompletely glottic closure presented during phonation (B). Postoperatively, the reinnervated left vocal fold (arrow) was nearly at the midline and appeared straight during inspiration (C). The bulk of left vocal fold (arrow) appeared the same as the right side postoperatively, and the glottic gap completely disappeared during phonation (D).

A total of 237 patients underwent preoperative and postoperative videostroboscopic examinations. Table 1 showed the preoperative and postoperative laryngeal appearance of this series. Preoperative video-recordings (see Supporting Information Video S1) showed mildly to severely bowing vocal fold edges, paramedian or intermediate vocal fold position, severely incomplete glottic closure and irregular vocal fold vibration during phonation in most patients (see Fig. 3A–B). Two years after laryngeal reinnervation, video-recordings (see Supporting Information Video S2) showed that 92.4% (219/237) of the patients had straight vocal fold edges, median or near median vocal fold position, symmetrical and regular vocal fold vibration, and complete glottic closure during phonation (see Fig. 3C–D). There was no vocal fold motion in paralyzed side pre- and post-operatively. No contradictory motion of vocal fold was observed postoperatively, and the postoperative glottic closure, vocal fold position, vocal fold edge, and phase symmetry and regularity were significantly improved compared to those of preoperation (*P*<0.001).

**Table 1:** Comparison of pre- and post-operative videostroboscopic findings in UVFP group.

	Preoperative	Postoperative	P value
Glottal Closure			<0.001
Complete	0	221	
Slightly incomplete	0	12	
Moderately incomplete	33	1	
Severely incomplete	204	3	
Vocal fold position			<0.001
Midline	11	168	
Paramedian	143	60	
Intermediate	68	8	
Lateral	15	1	
Vocal fold edge			<0.001
Straight	26	222	
Mildly bowing;	34	11	
Moderately bowing	76	3	
Severely bowing	101	1	
Phase symmetry			<0.001
Normal	0	219	
Mildly asymmetrical	0	14	
Moderately asymmetrical	36	2	
Severely asymmetrical	201	2	
Regularity			<0.001
Normal	0	220	
Mildly irregular	0	13	
Moderately irregular	42	2	
Severely irregular	195	2	

### Vocal function assessment

#### Perceptual evaluation and acoustic analysis

Preoperative and postoperative voice samples were available in 237 cases for perceptual evaluation and acoustic analysis. Significant improvements were also found in the overall grade, roughness, breathiness, asthenia and strain postoperatively (*P*<0.001, see Table 2). The inter-rater and intra-rater reliability was acceptable (inter-rater reliability >0.76, intra-rater reliability >0.81).

**Table 2:** Perceptual evaluation in UVFP group.

	n	Preoperative	Postoperative	Z	*P* Value
		Median(Q_L_, Q_U_)	Median(Q_L_, Q_U_)		
Grade	237	2.2 (2.0, 2.4)	0.0 (0.0, 0.4)	−13.404	<0.001
Roughness	237	1.8 (1.6, 2.0)	0.0 (0.0, 0.4)	−13.394	<0.001
Breathiness	237	2.0 (1.8, 2.4)	0.0 (0.0, 0.2)	−13.412	<0.001
Asthenia	237	1.4 (1.0, 2.0)	0.0 (0.0, 0.0)	−13.213	<0.001
Strain	237	1.0 (0.8, 1.4)	0.0 (0.0, 0.0)	−11.887	<0.001

Q_L_ = low quartile, Q_U_ = upper quartile.

Postoperative values of Jitter (local), Shimmer (local) and NHR in UVFP group were significantly lower than the corresponding preoperative values (*P*<0.001, see Table 3), and no significant differences were observed in the postoperative values between UVFP group and the control group (*P*>0.05, see Table 4). Furthermore, postoperative values of Jitter (local), Shimmer (local) and NHR in both genders of UVFP group also showed no significant differences compared with the corresponding gender in the control group (*P*>0.05, see Table 4).

**Table 3:** Acoustic analysis and maximum phonation time in UVFP group.

	n	Preoperative	Postoperative	Z	*P* Value
		Median(Q_L_, Q_U_)	Median(Q_L_, Q_U_)		
Jitter local (%)	237	1.52 (1.13, 2.04)	0.33 (0.21,0.58)	−13.346	<0.001
Shimmer local (%)	237	9.33 (7.95, 11.82)	4.22 (2.59, 6.42)	−13.346	<0.001
NHR (-dB)	237	0.147 (0.113, 0.212)	0.018 (0.012, 0.029)	−13.346	<0.001
MPT(second)	237	6.18 (4.89, 7.63)	17.22(14.44, 21.78)	−13.346	<0.001

NHR = mean noise to harmonics ratio, MPT = maximum phonation time, Q_L_ = low quartile, Q_U_ = upper quartile.

**Table 4:** Acoustic analysis and maximum phonation time between UVFP group and control group.

	Postoperative values in UVFP group		Control group		Z	*P* Value
	n	Median(Q_L_, Q_U_)	n	Median(Q_L_, Q_U_)		
Jitter local (%)	237	0.33 (0.21, 0.58)	237	0.32 (0.21, 0.53)	−0.936	0.349
Jitter local (female, %)	178	0.31 (0.21, 0.57)	178	0.30 (0.21, 0.52)	−0.454	0.650
Jitter local (male, %)	59	0.48 (0.19, 0.61)	59	0.39 (0.19, 0.56)	−1.109	0.267
Shimmer local (%)	237	4.22 (2.59, 6.42)	237	3.78 (2.46, 6.22)	−1.535	0.125
Shimmer local (female, %)	178	4.17 (2.78, 6.41)	178	3.45 (2.44, 6.18)	−1.592	0.111
Shimmer local (male, %)	59	4.86 (2.49, 6.49)	59	4.68 (2.49, 6.42)	−0.353	0.724
NHR (-dB)	237	0.018 (0.012, 0.029)	237	0.017 (0.012, 0.025)	−0.995	0.320
NHR (female, -dB)	178	0.018 (0.012, 0.028)	178	0.017 (0.012, 0.023)	−0.899	0.369
NHR (male, -dB)	59	0.019 (0.012, 0.034)	59	0.017 (0.012, 0.032)	−0.517	0.605
MPT(second)	237	17.22 (14.44, 21.78)	237	17.98(15.46, 21.98)	−1.540	0.124
MPT(female, second)	178	16.03 (14.25, 18.78)	178	16.70 (15.01, 19.26)	−1.563	0.118
MPT(male, second)	59	25.73 (20.54, 30.01)	59	26.12 (21.44, 30.43)	−0.431	0.667

NHR = mean noise to harmonics ratio, MPT = maximum phonation time, Q_L_ = low quartile, Q_U_ = upper quartile.

#### Maximum phonation time

As shown in Table 3, postoperative MPT values in UVFP group were significantly longer than the preoperative ones (*P*<0.001), and showed no significant difference compared with the values in the control group (*P*>0.05). Meanwhile, postoperative MPT value in both genders of UVFP group also showed no significant differences compared with the corresponding genders in the control group (*P*>0.05, see Table 4).

#### Eletromyographic findings

Preoperative and postoperative EMG results were available in 209 patients. The electrical activity during the EMG was divided into two types in the present study: spontaneous activity and voluntary motor-unit recruitment. To analyze the preoperative spontaneous activity in TA muscles of affected vocal folds at rest, the abnormal spontaneous activities such as positive waves, fibrillations or complex repetitive discharges were recorded in 17 patients at rest during 0.5–3 years after UVFP. Two years postoperatively, there was no evidence of abnormal spontaneous activities in the reinnervated TA muscles at rest.

In affected TA muscles during phonation with the greatest exertion, preoperative evaluation of voluntary motor-unit recruitment showed that there were 11 cases without motor unit potential, 147 cases with simple interference, 51 cases with mixed interference and no case with full interference. However, postoperative evaluation showed that there were 156 cases with full interference, 51 cases with mixed interference and 2 cases with simple interference. There was a significant improvement in postoperative recruitment in comparison with preoperative recruitment during phonation (*P*<0.001).

#### Postoperative complications

The postoperative complication incidence was 5.5% in the UVFP group (13 of 237 cases). Eleven of the 13 cases developed mild complications (ecchymosis in 5 patients, hematoma in 3 patients, wound infection in 1 patient, and other wound problems in 2 patients) postoperatively, which were resolved by symptomatic treatment within a few days. Two of 13 cases developed stridor and shortness of breath postoperatively due to hemorrhage, which is a severe complication and necessitates tracheotomy.

## Discussion

By now, laryngeal reinnervation falls mainly into following categories: 1) direct end-to-end anastomosis of RLN, 2) the free nerve grafting, 3) the neuromuscular pedicle, 4) nerve implantation, 5) hypoglossal nerve to RLN anastomosis, and 6) ansa cervicalis to RLN anastomosis. The main disadvantage of 1) and 2) is the possibility of laryngeal spasm due to misdirected regeneration of the abductor and adductor fibers. Our previous study demonstrated that nerve implantation, neuromuscular pedicle and neurorrhaphy achieved various degrees of reinnervation, with neurorrhaphy showing the best performance [16]. A single branch of ansa cervicalis was recommended to anastomose with RLN for treating UVFP by Crumley and other investigators [8], [9], [10], [11]. However, not all axons of nerve have the regenerative capacity to pass through the anastomosis site; nerve transfer requires a sufficient number of axons to reach the paralyzed laryngeal muscle and produce adequate reinnervation for muscular contraction [16]. Thus, Paniello recommended hypoglossal nerve as a donor nerve for anastomosis to RLN, because it has an advantage over Crumely's procedure, that is the hypoglossal nerve contains more axons than ansa cervicalis [19]. In this procedure, however, the length of RLN stump needs to be at least 3 cm for tension free anastomosis. Hypoglossal nerve-to-RLN anastomosis might be problematic when RLN is injured by thyroidectomy. Moreover, this procedure can also lead to hypoglossal nerve injury. Musings on the drawbacks of the above procedures call for novel treatment for laryngeal reinnervation. It was found that the loop of ansa cervicalis usually has a main branch, which contains 2–3 branches innervating the inferior portions of the sternohyoid, sternothyroid and omohyoid muscles [13], [14]. Additionally, fired electrical activity of all strap muscles was synchronous with the discharge of the TA muscle and increased with volume [20], [21]. These studies suggest that the main branch of ansa cervicalis may be an ideal candidate for laryngeal reinnervation.

In the present study, a significant increase in the density of motor unit potentials in TA muscles was observed postoperatively compared to that preoperatively during phonation. The density of motor unit potentials is the voluntary motor-unit recruitment which reflects the degree of innervation [22]. Thus postoperative EMG demonstrated that the affected laryngeal muscles were reinnervated successfully by the main branch of ansa cervicalis.

Postoperative videostroboscopy revealed that our procedure did not restore the functional motion of reinnervated vocal fold. Because the abductor and adductor fibers are distributed randomly in the RLN [23], the regenerated nerve fibers from ansa cervicalis extend along the endoneurial tube of the RLN in a random fashion, eventually innervating abductor and adductor muscles. Additionally, the ansa cervicalis only provides a physiologically quiet resting tone to its reinnervated muscles. This low-amplitude and weak neural excitation, compared with the large spikes and bursts in RLN motorneurones, can restore the reinnervated vocal fold bulk, tone and tension, rather than the functional motion [24]. Furthermore, adductor muscles are stronger than abductor muscle in the larynx [5], which may explain why reinnervated vocal folds are usually fixed at or near the midline, resulting in complete glottic closure postoperatively.

Postoperative perceptual evaluation, acoustic analysis and MPT showed that our procedure could provide normal or near-normal phonatory function, because it can restore physical properties of the vocal fold necessary for normal phonatory quality. The apposition of the symmetrical structures observed by videostroboscopy allowed the two vocal folds to oscillate synchronously during phonation, reducing aperiodicity and perturbation and producing a “pure” tone [24].

The satisfactory long-term outcomes of this procedure are theoretically attributed to the following characteristics: 1) the main branch of ansa cervicalis has a readily accessible location near the laryngeal complex, relatively constant resting tone, and little morbidity with its harvest [9], [11], [15]. Furthermore, it possesses more motor fibers than a single branch of ansa cervicalis, and can produces more adequate reinnervation [14]; 2) laryngeal muscle may still retain an ability to receive regenerating nerve axons even after long-term denervation [25], [26]. Our previous study revealed that within 2 years after denervation, the laryngeal muscles exhibited a persistent regenerative potential for delayed laryngeal reinnervation from the viewpoint of muscle stem cells [27]. These findings supported the feasibility of using delayed laryngeal reinnervation as a treatment of UVFP. However, theoretically, the longer the denervation period lasts, the poorer the outcome of the laryngeal reinnervation may be. Miyamaru et al. reported that the ability of TA muscle to receive nerve axons deteriorated in rats after an excessively long denervation period [25]. In the present study, most patients received our procedure within 2 years after nerve injury, which may also contribute to their satisfactory outcomes.

This procedure failed to restore satisfactory vocal function in 4 cases. In a 45-year old male, the length of distal RLN stump was only 1 cm, and the scarred tissue was not removed thoroughly from the nerve ends of distal RLN stump for tension-free anastomosis. The scarred tissue may block the axonal extension towards the targets, which was supported by the single recruitment of postoperative EMG. Another failure case was a 53-year old female who developed stridor and shortness of breath due to hemorrhage few hours postoperatively and necessitated tracheotomy. She exhibited no improvement in voice quality, and then received arytenoid adduction three years later. It was found that the distal RLN stump was completely disconnected from the main branch of ansa cervicalis, which can be caused by unexpected nerve injury during removal of the hematoma in emergency surgery. The last two cases showed insignificant improvement of voice quality after surgery. Their denervation course was beyond 3 years. The fixation of their cricoarytenoid joints was precluded preoperatively. Failure of phonatory function recovery was caused by insufficient laryngeal reinnervation after an excessively long denervation period. It is likely that a better outcome of reinnervation will be achieved if new axons reach the target muscles as soon as possible [28]. To date, there are still no definitive clinical data regarding the longest interval between the onset of UVFP and the nerve transfer allowing satisfactory physiological functional recovery of laryngeal muscles, which needs further research.

At present, UVFP remains a severe complication in thyroid gland surgery. The present study demonstrated that the main branch of ansa cervicalis-to-RLN anastomosis provides normal or near-normal phonatory functions. Based on our experiences, some surgical details of this procedure were described to help the surgeons: 1) the main branch of the ansa cervicalis usually overlies the common carotid artery or jugular vein, which can be easily found during operation; 2) RLN is constantly identified behind the cricothyroid joint and dissected retrogradely far enough to provide a tension-free anastomosis. It is easy and time-saving, without the need of exploring the injured area of RLN; 3) in case of insufficient length of distal extralaryngeal RLN stump, the lateral-inferior horn of the thyroid cartilage is exposed and the intralaryngeal RLN is dissected distally to add about 0.5–1 cm extra length for tension-free anastomosis; 4) the nerve ends of distal RLN stump should be prepared with resection of scarred tissue back to fasicles. It is critical to remove scarred or nonviable tissue from the nerve ends, which can be accomplished by direct inspection under the operating microscope; 5) suture material and manipulation of tissue, which can engender scarring, should be kept to a minimum. The nylon 11-0 threads used in this series is very thin so that the trauma caused by their insertion into the epineurium is minimized and induce no more than minimal tissue reaction, inasmuch as the reaction can disturb neurotization. Besides, the external epineurium can be gently peeled to allow separation and identification of fascicles. Care should be taken to avoid driving the needle or suture through the fascicles. The sutures should not be so snug that the ends of the nerve are strangulated or overlapping, which aims to bring the nerve fascicles into close apposition, not to achieve a watertight epineural closure [29]. For most patients, 3 sutures are enough for the anastomosis. However, after long time denervation, the RLN often become much thinner than the main branch of ansa cervicalis. To achieve a satisfactory end-to-end anastomosis, additional 1–2 sutures should be used to avoid the exposure of nerve fascicles at the anastomosis site; 6) in cases without main branches, two ansa cervicalis branches together-to-distal RLN stump anastomosis could be performed. Even if the ansa cervicalis on the RLN injury side is not available due to the primary surgery, contralateral ansa cervicalis can be used for nerve transfer (unpublished data). Taken together, ansa cervicalis main branch to RLN anastomosis offers the following advantages: 1) it can provide vocal fold tone, bulk and tension; 2) it does not alter the stiffness of vocal fold; 3) its technique is simple; 4) it does not preclude static methods (vocal fold injection, thyroplasty and arytenoid adduction) if it should fail. Meanwhile, the main disadvantage of the procedure is the time needed for ansa-RLN reinnervation as compared to other static medialization techniques [11], [24].

### Conclusions

Data from this study indicate that normal or near-normal phonatory quality may be achieved by delayed reinnervation with a main branch of ansa cervicalis anastomosis to RLN. It can be concluded that this procedure raises a successful reinnervation of laryngeal muscles and appears to be a feasible and effective approach in treating UVFP caused by thyroid surgery-associated recurrent laryngeal nerve injury.

## Supporting Information

Video S1 Preoperative videostroboscopic finding of a UVFP patient. The preoperative video-recording showed that mildly bowing vocal fold edges, paramedian vocal fold position, severely incomplete glottic closure and irregular vocal fold vibration during phonation in a UVFP patient.
(MPG)

Video S2 Postoperative videostroboscopic finding of the same UVFP patient. The postoperative video-recording showed that the patient had straight vocal fold edges, median vocal fold position, symmetrical and regular vocal fold vibration, and complete glottic closure during phonation.
(MPG)
